# Metabolic unhealthiness is an important predictor for the development of advanced colorectal neoplasia

**DOI:** 10.1038/s41598-017-08964-1

**Published:** 2017-08-21

**Authors:** Tae Jun Kim, Eun Ran Kim, Sung Noh Hong, Young-Ho Kim, Dong Kyung Chang, Jaehwan Ji, Jee Eun Kim, Hye Seung Kim, Kyunga Kim, Hee Jung Son

**Affiliations:** 1 Department of Medicine, Samsung Medical Center, Sungkyunkwan University School of Medicine, Seoul, Korea; 2 Center for Health Promotion, Samsung Medical Center, Sungkyunkwan University School of Medicine, Seoul, Korea; 3 Biostatistics and Clinical Epidemiology Center, Samsung Medical Center, Sungkyunkwan University School of Medicine, Seoul, Korea

## Abstract

Obesity is a well-known risk factor for colorectal neoplasia. Yet, the associations of both metabolic and obesity status with metachronous colorectal neoplasia remain unclear. We conducted a cohort study of 9,331 adults who underwent screening colonoscopy and surveillance colonoscopy. Participants were classified as metabolically healthy if they had no metabolic syndrome component. Participants were categorized into four groups according to body mass index and metabolic status: metabolically healthy non-obese (MHNO; n = 2,745), metabolically abnormal non-obese (MANO; n = 3,267), metabolically healthy obese (MHO; n = 707), and metabolically abnormal obese (MAO; n = 2,612). MAO individuals [n = 159 advanced colorectal neoplasia (AN) cases, 6.1%] and MANO individuals (n = 167 AN cases, 5.1%) had a higher incidence of AN compared with MHNO individuals (n = 79 AN cases, 2.9%). In a multivariable model, the risk of metachronous AN was higher in MANO (hazard ratio [HR] 1.43, 95% confidence interval [CI] 1.12–1.84) and MAO (HR 1.52, 95% CI 1.18–1.96) than in MHNO. In contrast, the risk of metachronous AN was not significantly elevated in MHO. In subgroup analyses, with or without adenoma at baseline, MAO was a risk group for metachronous AN, and MHO was not. Our findings suggest that metabolic unhealthiness is a significant predictor for metachronous AN.

## Introduction

The global epidemic of obesity continues to worsen, imposing considerable burdens on global health due to chronic diseases such as cardiovascular disease, type 2 diabetes, and some cancers^1–3^. As assessed via body mass index (BMI) and several other measures of adiposity, obesity has shown positive associations with colorectal cancer (CRC) and adenoma consistently^4–6^. However, most obese individuals do not develop CRC, indicating that the metabolic and other underlying pathophysiologic effects that accompany obesity vary across individuals and may have different implications for disease risk. Indeed, accumulating evidence suggests that obesity can be subdivided into distinct phenotypes based on the prevalence of metabolic abnormalities such as hypertension, dyslipidemia, and glucose intolerance, and that these phenotypes may be clinically relevant^7–10^.

Most obese individuals have one or more metabolic abnormalities. However, some obese individuals are metabolically healthy. Recent interest has focused on a unique subgroup of obese individuals who do not have metabolic abnormalities. In addition, non-obese individuals with metabolic abnormalities have been described^2, 11^. Previous studies on the effects of the metabolically healthy obese (MHO) phenotype on cardiovascular and neoplastic disease have yielded contradictory results^7, 12–16^. Likewise, recent studies concerning the association between colorectal neoplasia and these subgroups also showed contrary results^14, 16^. Therefore, we conducted a cohort study of incident colorectal neoplasia. We evaluated the risk of this disease according to both obesity and metabolic status. Furthermore, we employed a strict definition of metabolic health: individuals were only defined as being metabolically healthy if they did not have any metabolic parameters.

## Results

### Baseline characteristics of the study participants

The mean (standard deviation) age of the 9,331 study participants was 51.7 (6.8) years. The baseline characteristics of the participants are shown in Table 1 according to obesity and metabolic status (MHNO, MANO, MHO, and MAO). The obese groups (MHO and MAO) had higher proportions of men, current smokers, and modest alcohol consumers than the non-obese groups (MHNO and MANO). Participants in the metabolically unhealthy groups, MANO and MAO, were more likely to be older, aspirin users, and not to exercise regularly. These individuals had higher values for blood pressure, triglycerides, and fasting glucose, as well as lower levels of high-density lipoprotein cholesterol. However, no significant difference in family history of CRC was seen among the four groups.

**Table 1:** Baseline characteristics of study participants by obesity and metabolic status.

	MHNO (n = 2,745)	MANO (n = 3,267)	MHO (n = 707)	MAO (n = 2,612)	*P* value
Age, years	50.3 ± 6.3	52.6 ± 7.0	51.2 ± 6.6	52.1 ± 7.2	<0.001
Male	1,264 (46.1)	2,000 (61.2)	531 (75.1)	2,145 (82.1)	<0.001
BMI (kg/m^2^)	22.0 ± 1.8	22.7 ± 1.6	26.5 ± 1.4	27.0 ± 1.9	<0.001
Smoking status					<0.001
Never	1,740 (63.4)	1,698 (52.0)	297 (42.0)	908 (34.8)	
Former	600 (21.9)	886 (27.1)	256 (36.2)	1,001 (38.3)	
Current	405 (14.7)	683 (20.9)	154 (21.8)	703 (26.9)	
Modest alcohol intake consumption	249 (9.1)	568 (17.4)	153 (21.6)	710 (27.2)	<0.001
Regular exercise	978 (35.6)	1,047 (32.1)	259 (36.6)	815 (31.2)	<0.001
Family history of CRC	181 (6.6)	218 (6.7)	44 (6.2)	187 (7.2)	0.767
Aspirin use (%)	111 (4.0)	343 (10.5)	71 (10.0)	351 (13.4)	<0.001
SBP (mmHg)	108 (101–116)	121 (110–134)	113 (105–120)	124 (113–135)	<0.001
DBP (mmHg)	68 (61–74)	76 (68–84)	71 (65–77)	79 (71–86)	<0.001
Triglycerides (mg/dL)	78 (60–100)	116 (80–168)	92 (72–120)	145 (103–198)	<0.001
HDL-C (mg/dL)	61 (54–71)	49 (42–59)	54 (48–62)	45 (39–53)	<0.001
LDL-C (mg/dL)	119 (100–139)	123 (103–143)	127 (109–145)	126 (105–147)	<0.001
FBG (mg/dL)	86 (81–91)	93 (86–103)	88 (83–93)	97 (89–106)	<0.001

Values are expressed as means ± standard deviation, percentages, or median (interquartile range).

^a^High risk adenoma includes any adenoma larger than 1 cm, 3 or more adenomas, any adenoma with a villous component, or high-grade dysplasia.

MHNO, metabolically healthy non-obese; MANO, metabolically abnormal non-obese; MHO, metabolically healthy obese; MAO, metabolically abnormal obese; BMI, body mass index; CRC, colorectal cancer; SBP, systolic blood pressure; DBP, diastolic blood pressure; HDL-C, high-density lipoprotein cholesterol; LDL-C, low-density lipoprotein cholesterol; FBG, fasting blood glucose.

### The risk of metachronous colorectal neoplasia

Among the 9,331 participants, the incidence of any colorectal neoplasia and advanced neoplasia (AN) were 33.3% (3,109) and 4.7% (439) respectively. According to obesity (BMI criteria) and metabolic status, the incidences of any colorectal neoplasia were 26.8% (735/2,745) in MHNO, 34.4% (1,124/3,267) in MANO, 34.5% (244/707) in MHO, and 38.5% (1,006/2,612) in MAO. The incidences of AN were 2.9% (79/2,745) in MHNO, 5.1% (167/3,267) in MANO, 4.8% (34/707) in MHO, and 5.8% (159/2,612) in MAO. By sex, the incidences of any colorectal neoplasia were 23.9% (812/3391) in women and 38.7% (2,297/5,940) in men. The incidences of AN were 2.6% (89/3,391) in women and 5.9% (350/5,940) in men. The median follow-up period was 3.0 years (interquartile range, 2.7–3.3 years). Table 2 shows the results of multivariate Cox regression analyses for any colorectal neoplasia and for AN. All of the factors listed in the table were included as covariates in the multivariate regression analysis. The following factors showed significant associations with metachronous AN in this multivariate analysis: age (hazard ratio [HR] 1.05, 95% confidence interval [CI] 1.04–1.07), male sex (HR 1.45, 95% CI 1.10–1.92), current smoking (HR 1.76, 95% CI 1.34–2.30), and high risk adenoma at baseline colonoscopy (HR 3.03, 95% CI 2.48–3.69).

**Table 2:** The risk of metachronous colorectal neoplasia in multivariate analyses.

	Any colorectal neoplasia		Advanced colorectal neoplasia	
	aHR^a^ (95% CI)	*P* value	aHR^a^ (95% CI)	*P* value
Obesity and metabolic status				
MHNO (n = 2,745)	Reference		Reference	
MANO (n = 3,267)	1.15 (1.04–1.26)	0.005	1.43 (1.12–1.84)	0.005
MHO (n = 707)	1.12 (0.97–1.36)	0.121	1.39 (0.95–2.03)	0.108
MAO (n = 2,612)	1.21 (1.10–1.34)	<0.001	1.52 (1.18–1.96)	0.001
Age	1.03 (1.02–1.04)	<0.001	1.05 (1.04–1.07)	<0.001
Male sex	1.32 (1.19–1.48)	<0.001	1.45 (1.10–1.92)	0.009
Current smoking	1.31 (1.17–1.46)	<0.001	1.76 (1.34–2.30)	<0.001
Modest alcohol intake	1.12 (1.02–1.22)	0.017	1.11 (0.90–1.37)	0.333
Regular exercise	0.90 (0.84–0.98)	0.009	0.89 (0.74–1.07)	0.197
Regular aspirin use	1.05 (0.93–1.18)	0.425	1.21 (0.95–1.55)	0.13
Family history of CRC	1.10 (0.96–1.25)	0.168	1.06 (0.76–1.47)	0.753
High risk adenoma^b^ at screening colonoscopy	1.91 (1.74–2.10)	<0.001	3.03 (2.48–3.69)	<0.001

^a^Estimated from Cox proportional hazard models adjusted for age, sex, smoking status, alcohol intake, regular exercise, regular aspirin use, family history of colorectal cancer, and high risk adenoma at screening colonoscopy.

^b^High risk adenoma includes any adenoma larger than 1 cm, 3 or more adenomas, any adenoma with a villous component, or high-grade dysplasia.

^a^HR, adjusted hazards ratio; CI, confidence interval; MHNO, metabolically healthy non-obese; MANO, metabolically abnormal non-obese; MHO, metabolically healthy obese; MAO, metabolically abnormal obese; CRC, colorectal cancer.

Table 2 also shows the risk of any colorectal neoplasia and AN by metabolic status and obesity as defined based on BMI criteria (≥25 kg/m^2^). As compared with MHNO individuals, MANO individuals and MAO individuals had significantly increased risks of metachronous AN (MANO, HR 1.43, 95% CI 1.12–1.84; MAO, HR 1.52, 95% CI 1.18–1.96). In contrast, MHO individuals did not have a significantly increased risk of metachronous AN, as compared with MHNO individuals.

### Subgroup analyses of advanced colorectal neoplasia risk by presence of adenoma at baseline

To evaluate the consistency of the associations of obesity and metabolic status with metachronous AN, we performed subgroup analyses according to the presence of baseline adenoma, which is a most important factor affecting the risk of metachronous AN. The subgroup analyses did not show heterogeneities in the associations of obesity and metabolic status with the risk of metachronous AN, with no significant interactions by the presence of baseline adenoma (*P* for interaction = 0.309). In the subgroup with adenoma at baseline, MAO and MANO individuals had significantly increased risks of metachronous AN, in contrast, MHO did not have (Table 3). Even among subgroup without adenoma at baseline, MAO individuals compared with MHNO individuals had a significantly increased risk of metachronous AN, and MHO was not a risk group for metachronous AN. Additionally, using the abdominal obesity (waist circumference, ≥ 90 cm for men and ≥ 80 cm for women) criteria, the subgroup analyses showed consistent results (Table 4).

**Table 3:** The risk of metachronous advanced colorectal neoplasia by metabolic status and obesity using body mass index in subgroups with or without adenomas at baseline.

	Advanced colorectal neoplasia		
	Number of cases	aHR^a^ (95% CI)	*P* value
No adenoma at baseline			
MHNO (n = 1,642)	24	Reference	
MANO (n = 1,704)	42	1.23 (0.78–1.96)	0.374
MHO (n = 354)	10	1.50 (0.88–3.18)	0.113
MAO (n = 1,189)	41	1.73 (1.08–2.76)	0.023
One or more adenomas at baseline			
MHNO (n = 1,103)	55	Reference	
MANO (n = 1,563)	125	1.45 (1.08–1.95)	0.015
MHO (n = 353)	24	1.17 (0.74–1.83)	0.511
MAO (n = 1,423)	118	1.36 (1.01–1.84)	0.046

^a^Estimated from Cox proportional hazard models adjusted for age, sex, smoking status, alcohol intake, regular exercise, regular aspirin use, family history of colorectal cancer, and high risk adenoma at screening colonoscopy.

aHR, adjusted hazard ratio; CI, confidence interval; BMI, body mass index; MHNO, metabolically healthy non-obese; MANO, metabolically abnormal non-obese; MHO, metabolically healthy obese; MAO, metabolically abnormal obese.

**Table 4:** The risk of metachronous advanced colorectal neoplasia by metabolic status and obesity using the International Diabetes Federation waist circumference cut-points^a^ in subgroups with or without adenomas at baseline.

	Advanced colorectal neoplasia		
	Number of cases	aHR^a^ (95% CI)	*P* value
No adenoma at baseline			
MHNO (n = 1,550)	23	Reference	
MANO (n = 1,667)	40	1.36 (0.82–2.27)	0.237
MHO (n = 426)	11	1.76 (0.95–3.49)	0.068
MAO (n = 1,246)	43	1.90 (1.14–3.16)	0.014
One or more adenomas at baseline			
MHNO (n = 1,047)	49	Reference	
MANO (n = 1,543)	121	1.50 (1.09–2.08)	0.014
MHO (n = 403)	25	1.56 (0.97–2.49)	0.062
MAO (n = 1,449)	127	1.70 (1.23–2.35)	0.001

^a^International Diabetes Federation waist circumference cut-points: ≥ 90 cm for men and ≥ 80 cm for women.

^b^Estimated from Cox proportional hazard models adjusted for age, sex, smoking status, alcohol intake, regular exercise, regular aspirin use, family history of colorectal cancer, and high risk adenoma at screening colonoscopy.

aHR, adjusted hazard ratio; CI, confidence interval; BMI, body mass index; MHNO, metabolically healthy non-obese; MANO, metabolically abnormal non-obese; MHO, metabolically healthy obese; MAO, metabolically abnormal obese.

### The association between metabolic parameters and metachronous advanced colorectal neoplasia

We also evaluated the impact of the metabolic parameters on the risk of metachronous AN (Table 5). Other than waist circumference, we used the metabolic syndrome components, such as glucose, triglycerides, high-density lipoprotein-cholesterol, and blood pressure levels. In the Cox proportional hazard model adjusted for age, sex, BMI, smoking status, alcohol intake, regular exercise, regular aspirin use, family history of colorectal cancer, and high risk adenoma at screening colonoscopy, higher levels of fasting glucose and triglycerides were significant predictors of AN. These associations were evident in analyses using continuous variables, quartiles, or metabolic syndrome cutoff points for the metabolic variables. In contrast, high-density lipoprotein-cholesterol and systolic blood pressure did not show these associations consistently.

**Table 5:** The association between the metabolic parameters and metachronous advanced colorectal neoplasia.

	Univariate analysis			Multivariate analysis	
	HR (95% CI)	*P* value		HR (95% CI)	*P* value
By continuous variables					
Fasting glucose	1.015 (1.01–1.02)	<0.001		1.009 (1.004–1.014)	<0.001
Triglycerides	1.002 (1.001–1.003)	<0.001		1.001 (1.000–1.002)	0.033
HDL-C	0.984 (0.977–0.99)	<0.001		0.993 (0.986–1.000)	0.066
SBP	1.009 (1.006–1.013)	<0.001		1.004 (1.000–1.008)	0.091
By binary variables^a^					
Fasting glucose	1.53 (1.27–1.84)	<0.001		1.24 (1.03–1.50)	0.026
Triglycerides	1.59 (1.33–1.91)	<0.001		1.23 (1.02–1.49)	0.028
HDL-C	1.15 (0.94–1.40)	0.174		1.10 (0.90–1.35)	0.343
SBP	1.60 (1.34–1.93)	<0.001		1.18 (0.98–1.42)	0.089
**By quartiles**	Q1	Q2	Q3	Q4	*P* value
Fasting glucose					
Univariate model	Reference	1.21 (0.93–1.57)	1.33 (1.03–1.73)	1.86 (1.45–2.37)	<0.001
Multivariate model	Reference	1.10 (0.84–1.43)	1.18 (0.94–1.46)	1.37 (1.07–1.77)	0.039
Triglycerides					
Univariate model	Reference	1.12 (0.86–1.47)	1.34 (1.04–1.74)	1.85 (1.45–2.36)	<0.001
Multivariate model	Reference	1.02 (0.82–1.33)	1.18 (1.01–1.40)	1.33 (1.05–1.72)	0.029
HDL-C					
Univariate model	1.80 (1.39–2.32)	1.71 (1.31–2.22)	1.32 (1.01–1.74)	Reference	<0.001
Multivariate model	1.37 (1.05–1.79)	1.29 (1.02–1.66)	1.14 (0.86–1.50)	Reference	0.041
SBP					
Univariate model	Reference	0.98 (0.76–1.28)	1.15 (0.90–1.48)	1.68 (1.33–2.11)	<0.001
Multivariate model	Reference	0.97 (0.75–1.26)	1.01 (0.78–1.29)	1.17 (0.92–1.48)	0.395

Cox proportional hazard models adjusted for age, sex, body mass index, smoking status, alcohol intake, regular exercise, regular aspirin use, family history of colorectal cancer, and high risk adenoma at screening colonoscopy.

^a^Binary cutoff points: (1) high serum triglycerides ≥ 150 mg/dL; (2) low high-density lipoprotein-cholesterol ≤ 40 mg/dL for men and ≤ 50 mg/dL for women; (3) high systolic blood pressure ≥ 130 mmHg; (4) high fasting glucose > 100 mg/dL

HR, hazard ratio; CI, confidence interval; HDL-C, high-density lipoprotein cholesterol; SBP, systolic blood pressure.

## Discussion

In this cohort study, we found that non-obese individuals with metabolic abnormalities were at higher risks of colorectal neoplasia than were non-obese individuals without metabolic abnormalities. Our results also showed that MHO individuals, based on BMI, are not at an increased risk of colorectal neoplasia development, as compared with metabolically healthy non-obese individuals. In subgroup analyses, with or without adenoma at baseline, MAO was a risk group for metachronous AN, and MHO individuals was not at an increased risk of metachronous AN. The results were consistent either based on BMI or central obesity (by waist circumference). Our findings thus indicate that metabolically unhealthy status may be a more relevant etiological factor for the development of colorectal neoplasia than general obesity such as higher BMI.

Several previous studies have investigated the associations of obese/metabolic status with several outcomes, such as cardiovascular disease, type 2 diabetes, and neoplastic diseases (including colorectal adenoma and CRC)^13, 14, 16^. Some studies have suggested that the effects of the MHO phenotype are not enough to increase adverse outcomes. Meigs *et al*. reported that MHO individuals were not at increased risks of cardiovascular diseases or type 2 diabetes, as compared with metabolically healthy normal-weight individuals^13^. Recently, a nested study in the European Prospective Investigation into Cancer and Nutrition study revealed that MHO individuals had a similar CRC risk to metabolically healthy normal-weight individuals^14^. In contrast, a recent Korean cross-sectional study reported that MHO individuals are at increased risks of low-risk and high-risk colorectal adenomas, as again compared with metabolically healthy normal-weight individuals^16^. It is possible that these heterogeneous results are largely attributable to the various definitions of the MHO phenotype that have been used.

The common, principal criterion that has been used to identify MHO individuals is the absence of metabolic abnormalities (dyslipidemia, glucose intolerance, and hypertension)^8^. In this study, we also used a strict definition of metabolically healthy status: the absence of metabolic abnormalities. The metabolic factors are potential risk factors for colorectal neoplasia. However, the associations between these metabolic parameters, other than waist circumference, and colorectal neoplasia are inconsistent. Hypertension was found to be a risk factor for colorectal neoplasia in several studies, but conflicting results were reported in other studies^17–19^. Positive associations between overt diabetes or hyperglycemia and colorectal neoplasia were also observed in some studies, but not in others^20–23^. Previous studies on the association between dyslipidemia and colorectal neoplasia have also had inconsistent findings. The Metabolic Syndrome and Cancer Project (Me-Can), a large European cohort, reported a positive association between higher serum triglyceride levels and incident colorectal cancer, but this association was not confirmed in the European Prospective Investigation into Cancer and Nutrition (EPIC) study^24, 25^. In our results, glucose and triglycerides levels were significantly associated with incident AN after adjusting for potential confounding factors.

The mechanisms that link obesity and colorectal neoplasia are not fully established, although several possible mechanisms have been suggested^26–30^. Insulin resistance and hyperinsulinemia have been found to be involved in cellular mitogenic stimulation and to have anti-apoptotic activity that may be tumorigenic, as consequences of both their metabolic effects and the accumulation of visceral fat tissue^26–28^.– Adipose tissue-induced chronic inflammation is another potential mechanism for colon carcinogenesis. Instead of simply being fat storage, visceral adipose tissue is now recognized as an important endocrine organ that secretes numerous cytokines. Among the adipocytokines, leptin stimulates the growth of colonic epithelial cells and adiponectin is involved in carcinogenesis of the colon^29^. However, adipocytokines such as tumor necrosis factor, interleukin-6, plasminogen activator inhibitor, angiotensin, retinal binding protein-4, and resistin are mainly involved in hepatic gluconeogenesis, lipolysis, elevated blood pressure, and pro-thrombotic effects^30^. Consequently, these effects of the adipocytokines may contribute to the development of metabolic syndrome. Further research is needed to understand the underlying mechanisms of adipocytokines and their roles in colon carcinogenesis.

Several limitations need to be a considered when interpreting the results of our study. First, misclassifications might have occurred during the measurement of polyp size, and histological diagnosis. However, the colonoscopists and pathologists were unaware of the study objectives. These measurement errors would be non-differential and could possibly result in an underestimation of the association between metabolic/obese status and metachronous colorectal dysplasia. Second, we used BMI as a measure of obesity; however, BMI does not contain information on the composition and distribution of fat and muscle mass. If the MHO participants had a higher proportion of lean mass, then the association between the MHO phenotype and colorectal adenoma risk could have been attenuated. Third, although we carefully controlled for potential confounding factors in the multivariable analysis, we cannot exclude the possibility of residual confounding due to unmeasured covariates such as quality factors of colonoscopy. However, this study incorporated control measures for colonoscopic quality indicators, including bowel preparation, completeness of colonoscopy, and performance by an experienced gastroenterologist. Finally, this study focused on healthy participants who underwent a routine health check-up; thus, it may be difficult to generalize our findings to other populations.

This study also has several strengths. First, the cohort design of our study allowed us to identify temporal relationships, which is not usually possible in cross-sectional studies. Additional strengths include the use of high-quality, standardized clinical and laboratory methods, as well as the use of a strict definition of metabolically healthy status (no metabolic abnormalities).

In conclusion, this study showed that, a metabolic unhealthy state is associated with an increased risk of incident AN. Regardless of the presence of adenoma at baseline, the risk of metachronous AN was higher in MANO individuals. In contrast, the risk of colorectal neoplasia was not increased in MHO individuals. Therefore, the risk of advanced colorectal neoplasia appeared to have been mediated by metabolic parameters. Combinations of anthropometric measures with metabolic parameters may be useful for assessing the risks of metachronous colorectal neoplasia. Additionally, our study suggests that individuals who are metabolically unhealthy and have high risk adenomas at screening colonoscopy are at an elevated risk of metachronous AN. Surveillance colonoscopy should be recommended for these individuals.

## Methods

### Study population

**Figure 1:**
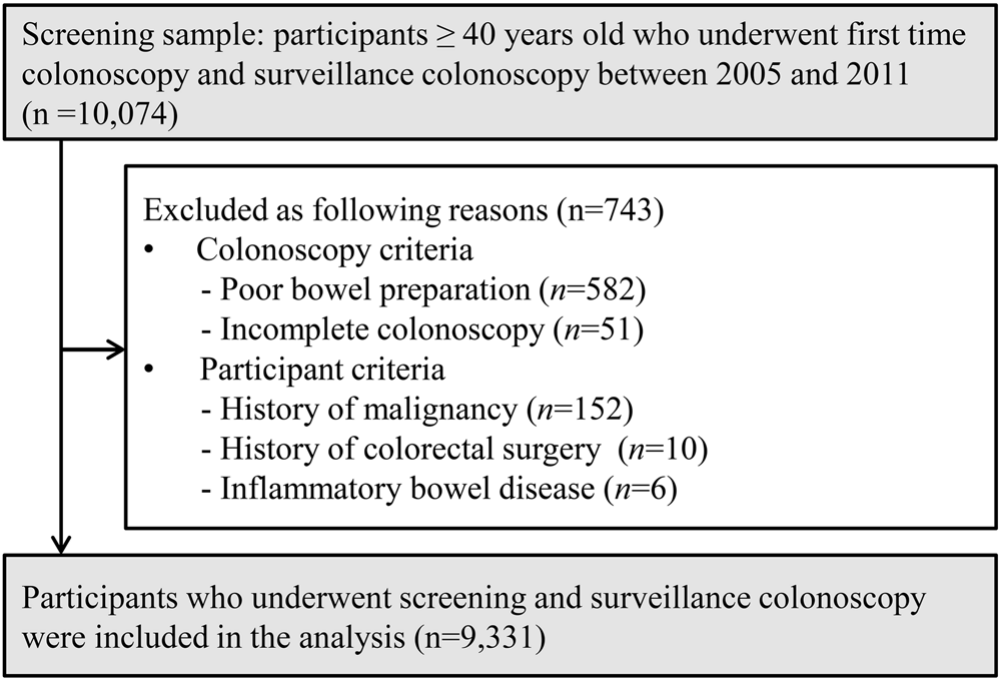
Flow diagram of study participants.

We conducted a retrospective cohort study of healthy participants, aged ≥ 40 years old, who underwent a routine health check-up that included colonoscopy at the Center for Health Promotion of Samsung Medical Center, South Korea, from January 2005 to December 2011^20^. Since our objective was to evaluate the association between obesity/metabolic status and metachronous colorectal neoplasia, we included participants who underwent a first-time colonoscopy for screening and a subsequent second colonoscopy for surveillance (n = 10,074). We excluded 743 participants who met any of the following exclusion criteria: poor bowel preparation (n = 582), incomplete colonoscopy (n = 51), history of malignancy including previous cancers and CRC detected at screening (n = 152), history of colorectal surgery (n = 10), and inflammatory bowel disease (n = 6). Finally, 9,931 asymptomatic healthy participants who underwent screening colonoscopy with or without polypectomy and surveillance colonoscopy were included in this study (Fig. 1). This study was approved by the Institutional Review Board of the Samsung Medical Center and was conducted in accordance with the Declaration of Helsinki and current legal regulations in Korea. The Institutional Review Board approval was obtained without specific informed consent because the study used only de-identified data that were collected for clinical purposes as part of the health screening check-up. However, informed consent was obtained from all subjects for their examinations at the health check-up.

### Data collection

The comprehensive health-screening program included anthropometric measurements, endoscopy, and serum biochemical measurements, as well as an epidemiological questionnaire on smoking habits, alcohol consumption, physical activity, medication history (including current use), personal medical history, and family history of cancer^20^. Personal medical history included diabetes mellitus, hypertension, dyslipidemia, cardiovascular disease, malignancy, colorectal neoplasia, and surgical history. Medication history included current and regular use of aspirin. Smoking status was divided into three groups: never, former, or current smoker. Alcohol consumption status was divided into mild (≤10 g/day) and modest (>10 g/day). Regular exercise was defined as exercising three or more times per week with moderate intensity activity. Weight and height were calculated to the nearest 0.1 kg and 0.1 cm, respectively, using an Inbody 720 machine (Biospace, Seoul, Korea). The participants’ weights and heights were measured with light clothing and bare feet. BMI was calculated as weight in kilograms divided by height in square meters (kg/m^2^). Blood pressure was measured in the seated position after > 5 minutes of quiet rest using an automated blood pressure monitor (Dinamap PRO 100; GE Healthcare, Milwaukee, Wisconsin).

After a ≥ 12 hour fast, fasting blood samples were collected in the morning and analyzed by the hospital clinical laboratory. Serum levels of glucose, total cholesterol, triglycerides, low-density lipoprotein-cholesterol, and high-density lipoprotein-cholesterol were measured. Total cholesterol, triglycerides, low-density lipoprotein-cholesterol, and high-density lipoprotein-cholesterol were measured using enzymatic colorimetric and liquid-selective detergent methods with a Hitachi 7600 (Hitachi, Tokyo, Japan). Serum glucose levels were measured using the hexokinase/glucose-6-phosphate dehydrogenase method with a Hitachi 7600 Modular Dp-110 autoanalyzer (Hitachi, Tokyo, Japan). The inter- and intra-assay coefficients of variation for quality control specimens were < 5% for all blood variables.

### Colonoscopies

Experienced board-certified gastroenterologists performed the colonoscopies using an colonoscope (Olympus CF-Q260AI or CF-Q260AL; Olympus Medical Systems, Tokyo, Japan) after bowel preparation with 4 L of polyethylene glycol solution (CoLyte and CoLyte-F; Taejun, Seoul, South Korea). The size of each lesion was estimated using an open biopsy forceps. The following information was recorded in the electronic medical record after the colonoscopy: number of polyps, locations and sizes of polyps, time and results of the last colonoscopy, family history of CRC, bowel preparation (excellent/good/fair/poor), cecal intubation time, withdrawal time, and completeness of the colonoscopy. Biopsy samples were sent to the pathology department, where qualified pathologists assessed the histopathology of the lesions^20^. AN was defined as any adenoma larger than 1 cm, any adenoma with a villous component, high-grade dysplasia, or invasive cancer.

### Definitions of obesity and metabolically healthy status

Obesity was defined according to Asian-specific criteria (BMI ≥ 25 kg/m^2^) or the International Diabetes Federation criteria (waist circumference ≥ 90 cm for men and ≥ 80 cm for women). Metabolically healthy status was defined as the absence of all of the following metabolic abnormalities at baseline:^8^ (1) high serum triglycerides, defined as ≥ 150 mg/dL (1.7 mmol/L) or drug treatment for this lipid abnormality (2) low high-density lipoprotein-cholesterol, defined as ≤ 40 mg/dL (1.0 mmol/L) for men and ≤ 50 mg/dL (1.3 mmol/L) for women or drug treatment for this lipid abnormality; (3) high blood pressure, defined as blood pressure ≥ 130/85 mmHg or drug treatment for previously diagnosed hypertension; (4) high fasting plasma glucose (FPG), defined as > 100 mg/dL (5.6 mmol/L) or drug treatment for previously diagnosed diabetes^8, 31^. As demonstrated by these criteria, we used a strict definition for metabolic healthy status which required that individuals had no metabolic abnormalities.

Study participants were classified into four groups based on BMI and metabolic status: (1) metabolically healthy non-obese (MHNO) group; (2) metabolically abnormal non-obese (MANO) group; (3) metabolically healthy obese (MHO) group; and (4) metabolically abnormal obese (MAO) group.

### Statistical analysis

Continuous variables are reported as means ± standard deviations, while categorical variables are presented as percentages. Continuous variables were compared between groups using Student’s *t*-test or the Kruskal-Wallis test, while categorical variables were compared using the Chi-squared test. Cox proportional hazard models were used to estimate hazard ratios (HRs) and 95% confidence intervals (CIs) for metachronous colorectal neoplasia in the MANO, MHO, and MAO groups using MHNO as the reference group. The multivariable model adjusted for age (/year), sex, smoking status (never vs. past vs. current smoker), alcoholic intake (mild vs. modest), regular exercise (yes vs. no), regular use of aspirin (yes or no), family history of CRC (yes or no), and high risk finding at screening colonoscopy (none vs. low risk adenoma vs. high risk adenoma).

We conducted subgroup analyses to identify interactions between obesity/metabolic status and clinically relevant groups, defined by the presence of baseline colorectal adenoma (No adenomas at baseline vs. one or more adenomas at baseline). Interactions between subgroups were tested using likelihood ratio tests comparing models with and without multiplicative interaction terms. A *P*-value < 0.05 was considered statistically significant. Statistical analyses were performed using SAS version 9.4 (SAS Institute, Cary, NC).

## References

[1] De Pergola G, Silvestris F (2013). Obesity as a major risk factor for cancer. J Obes.

[2] Renehan AG, Tyson M, Egger M, Heller RF, Zwahlen M (2008). Body-mass index and incidence of cancer: a systematic review and meta-analysis of prospective observational studies. Lancet.

[3] Vazquez G, Duval S, Jacobs DR, Silventoinen K (2007). Comparison of body mass index, waist circumference, and waist/hip ratio in predicting incident diabetes: a meta-analysis. Epidemiol Rev.

[4] Ben Q (2012). Body mass index increases risk for colorectal adenomas based on meta-analysis. Gastroenterology.

[5] Kitahara CM (2013). Prospective investigation of body mass index, colorectal adenoma, and colorectal cancer in the prostate, lung, colorectal, and ovarian cancer screening trial. J Clin Oncol.

[6] Okabayashi, K. *et al*. Body mass index category as a risk factor for colorectal adenomas: a systematic review and meta-analysis. *Am J Gastroenterol* **107**, 1175–1185; quiz 1186 (2012). 10.1038/ajg.2012.180 22733302

[7] Kramer CK, Zinman B, Retnakaran R (2013). Are metabolically healthy overweight and obesity benign conditions?: A systematic review and meta-analysis. Ann Intern Med.

[8] Primeau V (2011). Characterizing the profile of obese patients who are metabolically healthy. Int J Obes (Lond).

[9] Karelis AD (2008). Metabolically healthy but obese individuals. Lancet.

[10] Soriguer F (2013). Metabolically healthy but obese, a matter of time? Findings from the prospective Pizarra study. J Clin Endocrinol Metab.

[11] Wildman RP (2008). The obese without cardiometabolic risk factor clustering and the normal weight with cardiometabolic risk factor clustering: prevalence and correlates of 2 phenotypes among the US population (NHANES 1999-2004). Arch Intern Med.

[12] Arnlov J, Ingelsson E, Sundstrom J, Lind L (2010). Impact of body mass index and the metabolic syndrome on the risk of cardiovascular disease and death in middle-aged men. Circulation.

[13] Meigs JB (2006). Body mass index, metabolic syndrome, and risk of type 2 diabetes or cardiovascular disease. J Clin Endocrinol Metab.

[14] Murphy N (2016). A Nested Case-Control Study of Metabolically Defined Body Size Phenotypes and Risk of Colorectal Cancer in the European Prospective Investigation into Cancer and Nutrition (EPIC). PLoS Med.

[15] Voulgari C (2011). Increased heart failure risk in normal-weight people with metabolic syndrome compared with metabolically healthy obese individuals. J Am Coll Cardiol.

[16] Yun KE (2013). Impact of body mass index on the risk of colorectal adenoma in a metabolically healthy population. Cancer Res.

[17] Liu CS (2010). Central obesity and atherogenic dyslipidemia in metabolic syndrome are associated with increased risk for colorectal adenoma in a Chinese population. BMC Gastroenterol.

[18] Wong VW (2011). High prevalence of colorectal neoplasm in patients with non-alcoholic steatohepatitis. Gut.

[19] Kim JH (2007). Is metabolic syndrome a risk factor for colorectal adenoma?. Cancer Epidemiol Biomarkers Prev.

[20] Rampal, S. *et al.* Association between markers of glucose metabolism and risk of colorectal adenoma. *Gastroenterology* **147**, 78–87 e73 (2014). 10.1053/j.gastro.2014.03.006 24632359

[21] Kim BJ (2010). Clinical usefulness of glycosylated hemoglobin as a predictor of adenomatous polyps in the colorectum of middle-aged males. Cancer Causes Control.

[22] Wei EK (2006). C-peptide, insulin-like growth factor binding protein-1, glycosylated hemoglobin, and the risk of distal colorectal adenoma in women. Cancer Epidemiol Biomarkers Prev.

[23] Nishii T (2001). Glucose intolerance, plasma insulin levels, and colon adenomas in Japanese men. Jpn J Cancer Res.

[24] van Duijnhoven FJ (2011). Blood lipid and lipoprotein concentrations and colorectal cancer risk in the European Prospective Investigation into Cancer and Nutrition. Gut.

[25] Stocks T (2011). Metabolic factors and the risk of colorectal cancer in 580,000 men and women in the metabolic syndrome and cancer project (Me-Can). Cancer.

[26] Sandhu, M. S., Dunger, D. B. & Giovannucci, E. L. Insulin, insulin-like growth factor-I (IGF-I), IGF binding proteins, their biologic interactions, and colorectal cancer. *J Natl Cancer Inst* **94**, 972–980 (2002). 10.1093/jnci/94.13.972 12096082

[27] Wu, X., Fan, Z., Masui, H., Rosen, N. & Mendelsohn, J. Apoptosis induced by an anti-epidermal growth factor receptor monoclonal antibody in a human colorectal carcinoma cell line and its delay by insulin. *J Clin Invest* **95**, 1897–1905 (1995). 10.1172/JCI117871 PMC295734 7706497

[28] Wu Y, Yakar S, Zhao L, Hennighausen L, LeRoith D (2002). Circulating insulin-like growth factor-I levels regulate colon cancer growth and metastasis. Cancer Res.

[29] Hardwick JC, Ven Den Brink GR, Offerhaus GJ, Van Deventer SJ, Peppelenbosch MP (2001). Leptin is a growth factor for colonic epithelial cells. Gastroenterology.

[30] Mendonca FM (2015). Metabolic syndrome and risk of cancer: which link?. Metabolism.

[31] Alberti KG (2009). Harmonizing the metabolic syndrome: a joint interim statement of the International Diabetes Federation Task Force on Epidemiology and Prevention; National Heart, Lung, and Blood Institute; American Heart Association; World Heart Federation; International Atherosclerosis Society; and International Association for the Study of Obesity. Circulation.

